# MiR‐200c‐3p as a novel genetic marker and therapeutic tool for alopecia areata

**DOI:** 10.1111/srt.13639

**Published:** 2024-03-07

**Authors:** Ahmed Ibrahim AbdElneam, Mohammed Saleh Al‐Dhubaibi, Saleh Salem Bahaj, Ghadah Alhetheli

**Affiliations:** ^1^ Department of Clinical Biochemistry Department of Basic Medical Sciences College of Medicine Shaqra University Dawadmi Saudi Arabia; ^2^ Molecular Genetics and Enzymology Department Human Genetics and Genome Research Institute National Research Center Dokki, Cairo Egypt; ^3^ Departments of Dermatology College of Medicine Shaqra University Dawadmi Saudi Arabia; ^4^ Department of Microbiology and Immunology Faculty of Medicine and Health Sciences Sana'a University Sanaa Yemen; ^5^ Divisions of Dermatology and Cutaneous Surgery College of Medicine Qassim University Buraydah Saudi Arabia

**Keywords:** alopecia areata, EGFR, miR‐200c‐3p, PLCG1, RPS6KP1

## Abstract

**Background:**

MicroRNAs (miRNAs) are small RNA molecules that regulate gene expression in diverse biological processes. They hold promise as therapeutic candidates for targeting human disease pathways, although our understanding of their gene regulatory mechanism remains incomplete. Alopecia areata (AA) is a prevalent inflammatory ailment distinguished by the infiltration of T cells targeting the anagen‐stage hair follicles. The scarcity of effective remedies for AA may stem from limited understanding regarding its precise cellular mechanism.

**Aim:**

To investigate and examine the importance and role of the miR‐200c‐3p as a genetic indicator for AA, and its possible impact on disease progression.

**Subjects and methods:**

Case‐control study included 65 patients with AA and 65 matched healthy controls. A real‐time PCR technique was used to measure the expression of miR‐200c‐3p for both groups. Bioinformatic tools were used for prediction with genes and gene‐gene interaction, and protein‐protein interaction.

**Results:**

The expression levels of miR‐200c‐3p were significantly higher in AA patients than in healthy controls. We predicted that miR‐200c‐3p plays a markable role in the development of AA by its effect on the EGFR tyrosine kinase inhibitor resistance pathway.

**Conclusion:**

We were able to identify the influence of miR‐200c‐3p on both PLCG1 and RPS6KP1 genes which in turn regulate the EGFR tyrosine kinases resistance pathway that displayed the most substantial increase in activity. Our outcomes shed light on the era of the potential theranostic role of this innovative miRNA in AA.

**Abbreviations**
adenylate‐uridylate (AU)‐rich elements: AREs
alopecia areata: AA
area under the curve: AUC
dermal papilla cells: DPCs
hair follicles: HFs
hair loss: HL
MicroRNAs: miRNAs
protein‐protein interaction: PPI

## 1 INTRODUCTION

Alopecia areata (AA) is an autoimmune condition characterized by the loss of hair, which is not accompanied by any scarring of the scalp. This disease manifests with sudden and unpredictable hair loss (HL), affecting individuals in a non‐uniform manner, with hair falling out in patches or, in some cases, leading to complete baldness. The underlying cause of AA stems from the body's immune system mistakenly targeting and attacking the hair follicles (HFs). Consequently, this immune response results in the unfortunate consequence of HL, leaving affected individuals with an altered appearance and potential psychological impact.^1^ It is the second most common cause of HL worldwide. The condition can affect both males and females of any age, although it often begins in childhood or early adulthood.^2^

The precise etiology of AA has yet to be comprehensively comprehended, however, it is postulated to be intricately intertwined with a confluence of genetic, environmental, and immunological elements. The existence of a familial lineage characterized by this disorder is regarded as a consequential determinant, thereby implying the existence of a hereditary predisposition.^3^

According to the degree of HL, AA can be classified into three distinct categories: patchy alopecia, characterized by partial HL on the scalp; alopecia totalis, which involves complete HL on the scalp; and alopecia universalis, which results in HL on both the scalp and body. Various clinical manifestations of scalp HL can be observed, including patchy forms that are either localized or multiple, ophiasis, sisaipho, reticulate, and diffuse patterns.^4^

The breakdown of the immunological privilege of HFs, which causes an increase in self‐peptide/MHC expression throughout the follicular epithelium, is thought to be the first step toward the beginning of the disease. The infiltration of purportedly self‐reactive T lymphocytes into the HF is linked to HL. This mechanism is assumed to tilt the microenvironment of the HFs toward active inflammation rather than the normally homeostatic immunological state.^5^

The dearth of reliable biomarkers and effective therapeutic interventions for AA might be attributed to a limited understanding of its precise cellular mechanism. In recent years, significant strides have been made in unraveling the pathogenesis of AA, yielding fruitful outcomes in the form of several promising biomarkers that are closely associated with disease severity, activity, response to treatment, and prognosis. These advancements have not only shed light on the intricate workings of AA but have also paved the way for potential breakthroughs in the diagnosis, management, and prediction of outcomes for individuals affected by this condition.^6^, ^7^, ^8^

Micro‐RNAs (miRNAs), also known as non‐coding RNA molecules, are small molecules that are essential for the control of genes. The pathophysiology and development of AA have been linked to miRNAs in this regard. In AA, the skin of the scalp and HFs have been found to have dysregulated miRNAs in a number of investigations.^9^

In the realm of scientific inquiry, a triad of comprehensive investigations was undertaken to meticulously scrutinize and evaluate the intricate miRNA expression profiles present within the cellular milieu of the dermal papilla cells (DPCs). These studies focused their attention on individuals afflicted with androgenetic alopecia, a condition characterized by the gradual and progressive loss of hair, as well as on the DPCs of healthy individuals. It is worth noting that these meticulous analyses were conducted with the addition of a treatment modality, which adds a layer of complexity and intrigue to the investigations at hand.^10^, ^11^, ^12^

Recent investigations conducted by Wang et al have uncovered noteworthy findings regarding the variable expression patterns of miRNAs within the afflicted dermal tissue of the murine C3H/HeJ model of AA, which specifically aim at modulating immune‐regulatory pathways. Nevertheless, the current body of literature concerning the quantification of diverse miRNA levels within both blood and tissue samples obtained from individuals suffering from AA remains limited and inconclusive.^13^

It is believed that these dysregulated miRNAs affect several cellular functions, such as the HF cycle, inflammation, and the immune system. MiRNAs have the ability to regulate the expression of important components involved in hair development and maintenance by targeting particular genes.^14^

In our population, several studies have been conducted to understand the relationship between genetic factors and dermatological conditions.^15^, ^16^, ^17^, ^18^, ^19^, ^20^, ^21^, ^22^, ^23^, ^24^ However, according to our knowledge, no study tries to determine the mechanisms by which miRNAs contribute to AA.

### 1.1 Aim of the study

The primary objective of our research is to explore and analyze the significance and function of the MicroRNA named ‘hsa‐miR‐200c‐3p’ on the development of AA by identifying the impact of hsa‐miR‐200c‐3p expression on the pathway involved in AA, and to elucidate the intricate relationship between this specific miRNA and the development and progression of AA. At the same time, we intended to establish it as a groundbreaking and innovative diagnostic approach. By delving into this pathway, we seek to uncover crucial insights and valuable knowledge that can potentially contribute to a deeper understanding of AA and pave the way for more effective treatment modalities.

## 2 SUBJECTS AND METHODS

### 2.1 Subjects

This is a case‐control study that involved 65 patients with AA and 65 age and sex‐matched healthy controls. The study was approved by the Ethics Committee of Shaqra University with agreement reference number *ERC_SU_202300031* and was carried out in accordance with the Declaration of Helsinki's rules. All participants were given informed written consent.

All participants were subjected to detailed medical history which included information on demographic characteristics, medical history, and medication use.

Inclusion Criteria: Patients with a clinically approved diagnosis of AA (patchy or ophiasis), regardless of gender or age, and those who were off therapy in the past three months are eligible.

Exclusion Criteria: Alopecia totalis, alopecia universalis, and other causes of alopecia like telogen effluvium, tinea capitis, and trichotillomania. Taking vitamin D supplements or other vitamins for the past six months. Patients with a documented vitamin D deficiency. Pregnancy or breastfeeding. Patient with any other autoimmune disease.

### 2.2 RNA extraction and RT‐PCR

Total plasma miRNAs were isolated using Direct‐zol™ RNA MiniPrep (Zymo Research) according to the manufacturer's protocol. Each RNA sample was quantified with a spectrophotometer (NanoDrop 1000, Thermo Scientific, Wilmington, Delaware). Isolated miRNAs were converted to complementary DNA (cDNA) by miRCURY LNA RT kit (Qiagen). qRT‐PCR reactions were conducted with the cDNA and miRCURY LNA SYBR Green PCR Kit (Qiagen) (contain primer for hsa‐miR‐200c‐3p and housekeeping gene) using a Bio‐Rad iCycler (Bio‐Rad Laboratories, Hercules, CA). Quantitative real‐time polymerase chain reactions will be performed in duplicate for all samples. qRT‐PCR data analysis: Relative gene expression level was calculated by 2^−(ΔΔCt)^ equation.

### 2.3 Target genes analysis

To better understand the biological function of the significant dysregulated miRNAs, their putative target genes were predicted under a confidence interval of 95% using TargetScan (https://www.targetscan.org/vert_80/), miRDB (https://mirdb.org/), MiRTarBase9.0 (https://mirtarbase.cuhk.edu.cn/), and miRWalk databases (http://mirwalk.umm.uni‐heidelberg.de/); to gain deep insight into the biological functions of the differential miRNAs and target genes, the pathways of target genes will be analyzed by KEGG database and protein‐protein interaction (PPI) enrichment *p*‐value was computed by STRING tool using Shiny Go 0.77 web site (http://bioinformatics.sdstate.edu/go/)

### 2.4 tatistical analysis

SPSS software version 17 was used to analyze the data. ROC curve analysis was used to evaluate the accuracy of hsa‐miR‐200c‐3p. The best cutoff values were selected based on maximum sensitivity and specificity for prediction. The area under the curve (AUC) criteria was used to qualify the accuracy. Two‐sided tests were conducted with a significance level of 0.05. Independent *t*‐tests, chi‐square tests, and One‐way ANOVA tests were used to compare groups and examine relationships between data.

## 3 RESULTS

### 3.1 Clinical and demographic data for patients and controls

**FIGURE 1:**
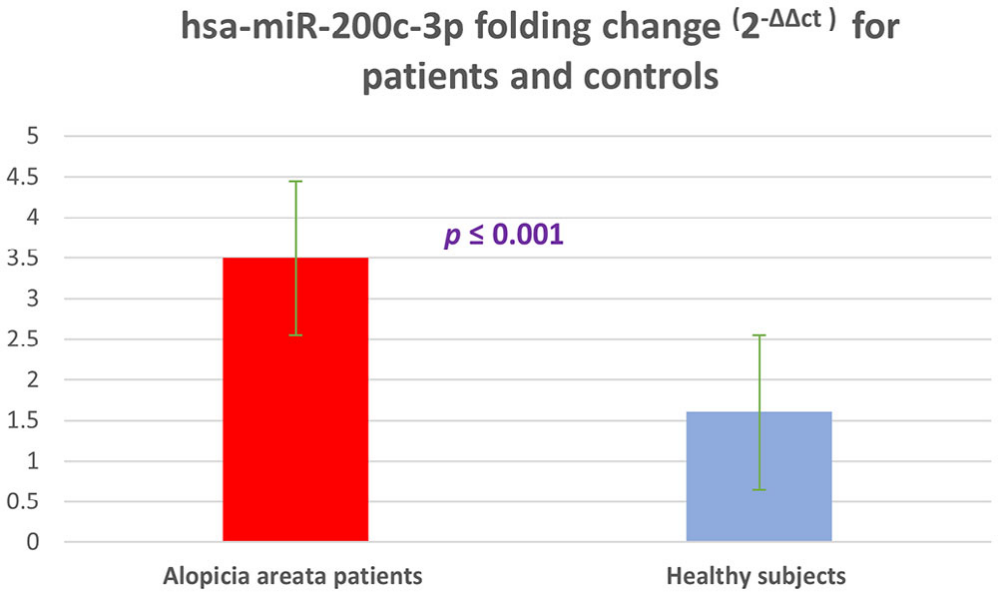
The difference in hsa‐miR‐200c‐3p folding change (2^‐∆∆ct^) for AA patients and healthy controls.

Clinical and demographic data of both patients and matched healthy control groups are presented in Table 1. It is worth noting that there is a significant difference in the hsa‐miR‐200c‐3p expression between the patients and controls, with the patients displaying a higher level of expression (*p* = 0.001). This suggests a potential correlation between this specific MicroRNA and AA in these individuals, thereby pointing toward a possible role of hsa‐miR‐200c‐3p in the development or progression of AA. This finding is further supported by the visual representation provided in Figure 1, which highlights the discrepancy in expression levels between the two groups.

**TABLE 1:** Clinical and laboratory characteristics for AA patients and healthy controls.

Variables	AA patients *N* = 65	Healthy controls *N* = 65	*p*‐Value
Age (mean ± SD)	23.7 ± 2.6	22.8 ± 3.9	0.886
Male (N/%)	35(53.8%)	27(41.5%)	0.432
Female (N/%)	30(46.2%)	38(58.5%)	0.552
AA type			
Localized patchy	30(46.2%)	—‐	NA
Multiple patchy	25 (38.4%)	—‐	NA
Ophiasis	10 (15.4%)	—‐	NA

Abbreviations: %, percentage; AA, alopecia areata; N, number; NA, not applied; SD, standard deviation.

### 3.2 The alternation at hsa‐miR‐200c‐3p expression in AA patients

The diagnostic potential of hsa‐miR‐200c‐3p expression in disease identification exhibits a remarkably high level of precision, as exemplified by the impressive value of AUC, which stands at 0.885. This value serves as a testament to the robustness of the test, as it possesses a formidable capability to discern between individuals afflicted with the disease and those who are not. The confidence interval (CI) associated with this prediction, spanning from 80.5% to 96.6%, further reinforces the reliability of these findings, as it indicates a profound level of certainty in the accuracy of the test. Moreover, the *p*‐value linked to this prediction, amounting to 0.001, offers supplementary evidence regarding the significance of the results, signifying that the likelihood of obtaining such outcomes by mere chance is exceedingly low.

**FIGURE 2:**
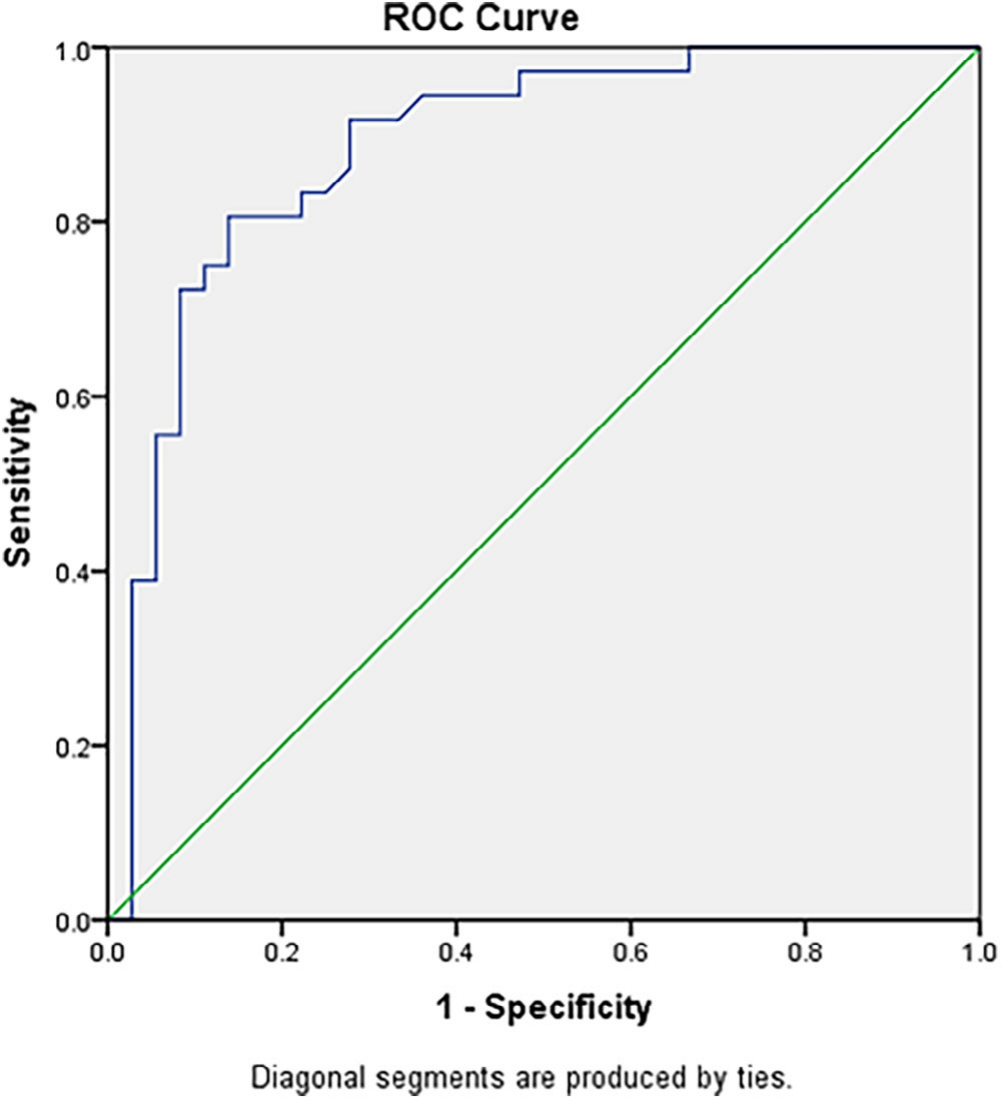
ROC curve analysis for hsa‐miR‐200c‐3p expression.

In order to achieve this exceptional level of diagnostic accuracy, a specific threshold value of 1.8 for hsa‐miR‐200c‐3p expression was employed. This signifies that individuals surpassing this threshold were categorized as possessing the disease, while those falling below it were labeled as non‐afflicted. The sensitivity of this prediction, which gauges the proportion of correctly identified true positive cases as determined by the test, was established at 80.5%. This indicates that the test successfully detected the presence of the disease in a substantial majority of cases, precisely 86.1% of cases. The presence of such a heightened sensitivity suggests that the test exhibits a minimal rate of false negatives, thereby minimizing the likelihood of overlooking instances of the disease (Figure 2).

### 3.3 Compare the alternation in hsa‐miR‐200c‐3p expression among different AA types

**FIGURE 3:**
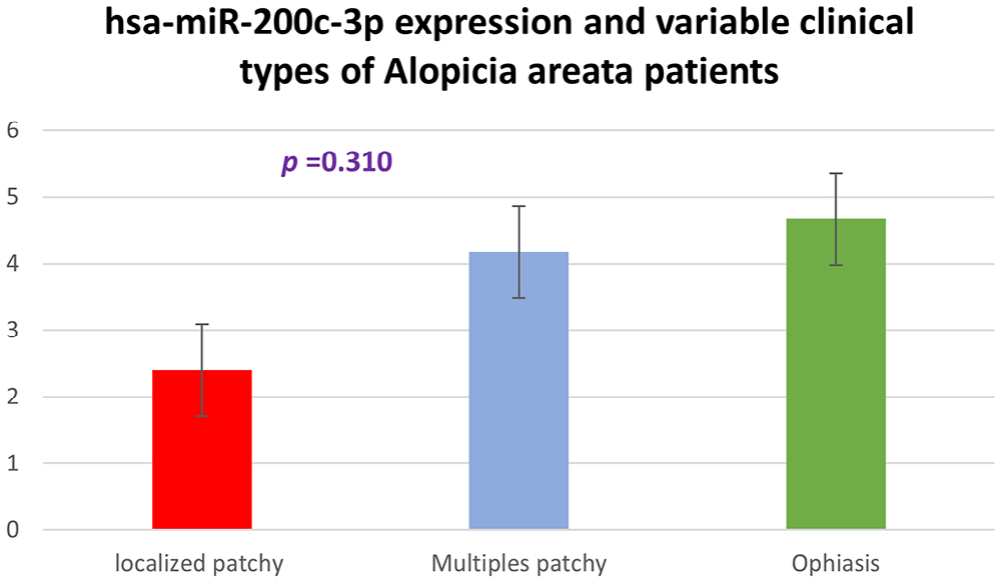
Comparison of hsa‐miR‐200c‐3p expression among variable clinical types of AA patients.

There is a difference in the hsa‐miR‐200c‐3p folding expression among individuals afflicted with ophiasis, as opposed to those suffering from localized or multiple patchy AA, but without significant variation (*p *= 0.310) (Figure 3).

## 4 BIOINFORMATIC ANALYSIS RESULTS

### 4.1 Prediction of target genes for the expression of hsa‐miR‐200c‐3p

To investigate the potential target genes of the differentially expressed miRNAs, an analysis was conducted using the miRWalk tool. This comprehensive analysis involved the utilization of various target prediction algorithms, such as TargetScan, mirDB, and MirTarBase. The aim was to classify and annotate the hsa‐miR‐200c‐3p within three distinct genomic regions, namely the 3′UTR, 5′UTR, and CDS. The selection of these target genes was based on a set of specific criteria, including a high binding probability, low binding energy, and the presence of Adenylate‐uridylate (AU)‐rich elements (AREs).

Additionally, the position of the miRNA attachment on the target gene (i.e., 3′UTR, 5′UTR, or CDS) was also considered. To ensure a comprehensive analysis, the hsa‐miR‐200c‐3p was evaluated using multiple target prediction tools, including miRWalk, TargetScan, mirDB, and MirTarBase. Based on the aforementioned criteria and analysis, there were six genes within the 3′UTR region identified as potential targets of hsa‐miR‐200c‐3p. Those six genes were: RPS6KB1, CDH11, DNAJC3, DNMT3B, NLGN4X, and SLC1A2.

Additionally, one gene, SLC1A2, was found to be targeted by hsa‐miR‐200c‐3p within the 5′UTR region. Furthermore, four genes (GLI3, FN1, ZNF217, and PLCG1) were identified as potential targets of hsa‐miR‐200c‐3p within the coding sequence (CDS) region. The complete list of predicted target genes can be found in Table 2.

**TABLE 2:** Predicted target genes for hsa‐miR‐200c‐3p by bioinformatics tools.

Gene	Reference sequence ID	Binding probability	Binding energy	AU‐rich region fraction	Position	Binding site
RPS6KB1	NM_003161	1.00	−19.424	0.56	3′UTR	2502,2519
CDH11	NM_001797	1.00	−18.079	0.44	3′UTR	6155,6169
DNAJC3	NM_006260	1.00	−17.846	0.56	3′UTR	4215,4230
DNMT3B	NM_001207056	1.00	−15.567	0.56	3′UTR	2923,2971
NLGN4X	NM_020742	1.00	−15.491	0.54	3′UTR	3068,3086
SLC1A2	XM_017018137	1.00	−14.401	0.52	3′UTR	4078,4096
SLC1A2	XM_047427436	1.00	−6.748	0.52	5′UTR	420,437
GLI3	XM_047420205	1.00	−18.079	0.35	CDS	2757,2771
FN1	NM_212482	1.00	−17.159	0.43	CDS	4711,4727
ZNF217	NM_006526	1.00	−15.567	0.6	CDS	2493,2524
PLCG1	NM_002660	1.00	−14.908	0.48	CDS	1347,1366

Abbreviations: AU, Adenylate‐uridylate.; CDH11, cadherin 11; CDS, coding sequence; DNAJC3, DnaJ heat shock protein family (Hsp40) member C3; DNMT3B, DNA methyltransferase 3 beta; FN1, fibronectin 1; GLI3, GLI family zinc finger 3; NLGN4X, neuroligin 4 X‐linked; PLCG1, phospholipase C gamma 1;RPS6KB1, ribosomal protein S6 kinase B1; SLC1A2, solute carrier family 1 member 2; UTR, untranslated region; ZNF217, zinc finger protein 217.

### 4.2 Predicted pathways targeted of selected genes

**FIGURE 4:**
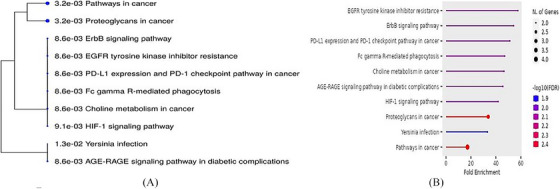
A dot‐plot was performed using Shiny GO 0.77 resource to represent the outcomes of KEGG pathway enrichment analyses carried out on and its validated pathways. (A) A hierarchical clustering tree summarizes the correlation among significant pathways targeted genes identified in this study. Pathways with many shared genes are clustered together. Bigger dots indicate more significant *p*‐values. (B) Fold enrichment showing the percentage of genes belonging to a particular pathway. Pathways have been filtered based on the FDR cutoff the top 10 most significant pathways are shown here.

To comprehend the biological implications of various selected gene expressions and the potential impact on the development of AA, the pathway for the genes identified in this study was predicted. Moreover, an investigation was carried out to shed light on the involvement of hsa‐miR‐200c‐3p in these selected genes. To achieve this objective, an enrichment analysis was conducted, along with a KEGG pathway analysis, to ascertain the transcriptional gene regulation, as depicted in Figure 4.

An intriguing observation was made during this analysis, revealing the participation of genes PLCG1 and RPS6KB1 in a total of 5 KEGG pathways, with PLCG1 being involved in 10 pathways. Additionally, it was duly noted that the fold enrichment of EGFR tyrosine kinase inhibitor resistance (hsa01521) was significantly higher compared to the aforementioned 10 pathways, as indicated in Table 3. This finding may potentially signify the crucial role played by genes: PLCG1, RPS6KB1, and EGFR tyrosine kinase inhibitor resistance in the development of AA.

**TABLE 3:** KEGG pathways and genes involved in selected genes.

KEGG pathway predicted	Fold enrichment	Genes identified in the study associated with KEGG pathway	Enrichment FDR
EGFR tyrosine kinase inhibitor resistance (hsa01521)	57.7	RPS6KB1 and PLCG1	8.6E‐03^*^
ErbB signaling pathway (hsa04012)	54.3	RPS6KB1 and PLCG1	8.6E‐03^*^
PD‐L1 expression and PD‐1checkpoint pathway in cancer (hsa05235)	51.2	RPS6KB1 and PLCG1	8.6E‐03^*^
Fc gamma R‐mediated phagocytosis (hsa04666)	47	RPS6KB1 and PLCG1	8.6E‐03^*^
Choline metabolism in cancer (hsa05231)	46.5	RPS6KB1 and PLCG1	8.6E‐03^*^
AGE‐RAGE signaling pathway in diabetic complications (hsa04933)	45.6	FN1 and PLCG1	8.6E‐03^*^
HIF‐1 signaling pathway (hsa04066)	41.8	RPS6KB1 and PLCG1	9.1E‐03^*^
Proteoglycans in cancer (hsa05205)	33.9	RPS6KB1, FN1, and PLCG1	3.2E‐03^*^
Yersinia infection (hsa05135)	33.3	FN1 and PLCG1	1.3E‐02^*^
Pathways in cancer (hsa05200)	17.2	GLI3, RPS6KB1, FN1, and PLCG1	3.2E‐03^*^

Abbreviations: CDH11, cadherin 11; DNAJC3, DnaJ heat shock protein family (Hsp40) member C3; DNMT3B, DNA methyltransferase 3 beta; FN1, fibronectin 1; GLI3, GLI family zinc finger 3; KEGG, Kyoto Encyclopedia of Genes and Genomes.; NLGN4X, neuroligin 4 X‐linked; PLCG1, phospholipase C gamma 1;RPS6KB1, ribosomal protein S6 kinase B1; SLC1A2, solute carrier family 1 member 2; ZNF217, zinc finger protein 217.

* Highly significant differences, *p* ≤ 0.001.

### 4.3 Functional annotation of differentially expressed genes and PPI

**FIGURE 5:**
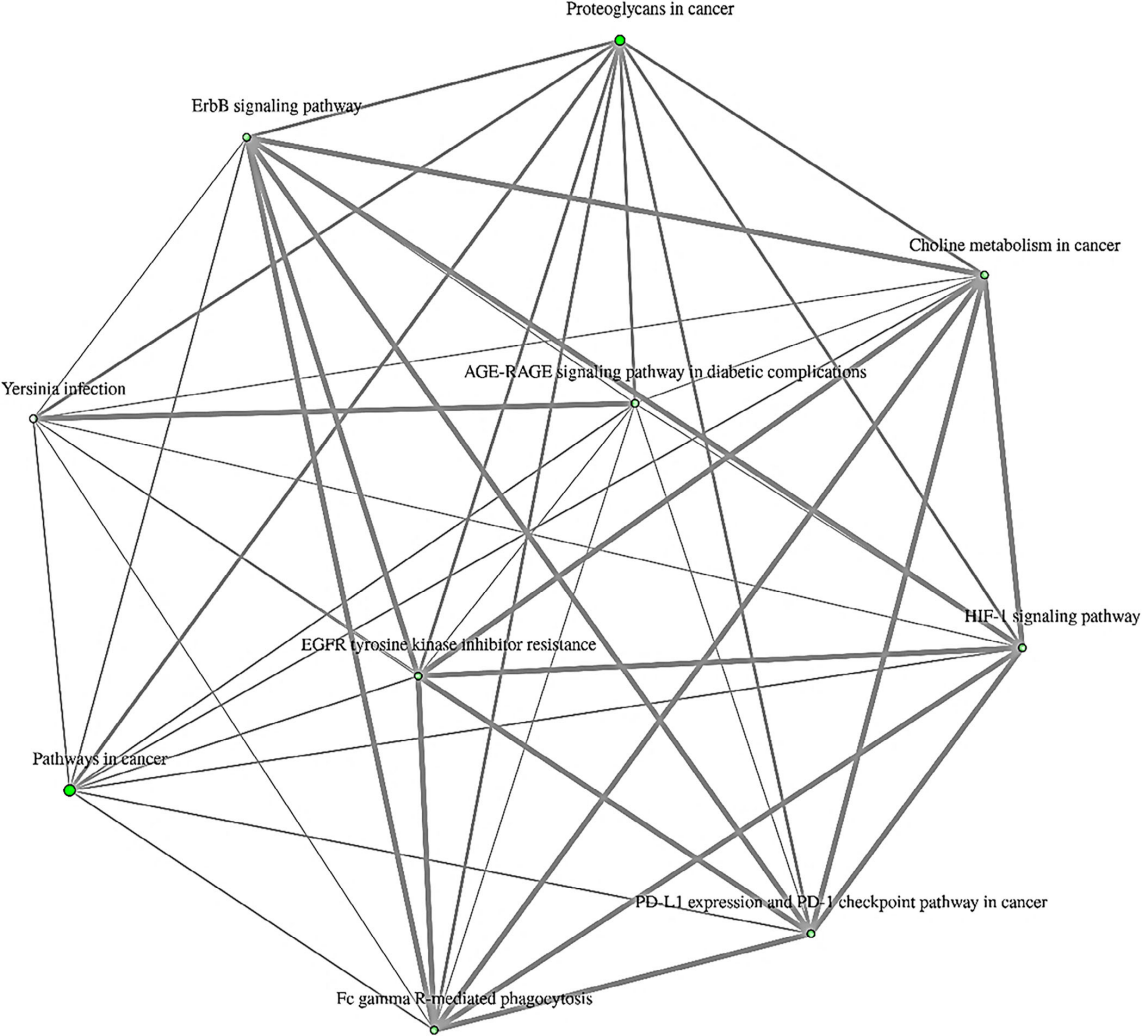
Functional annotation networks. The interactive plot also shows the relationship between enriched gene ontology and enriched pathways using ShinyGO 0.77 software. Two pathways (nodes) are connected if they share 20% (default) or more genes. Darker nodes are more significantly enriched gene sets. Bigger nodes represent larger gene sets. Thicker edges represent more overlapped genes.

To elucidate the specific role played by hsa‐miR‐200c‐3p in the biological processes affected by the differentially expressed genes identified in this particular study, a comprehensive analysis of functional annotation networks was conducted. The resultant interconnected network, which is visually represented in Figure 5, showcases all the functionally significant terms that are associated with the 10 pathways under investigation.

Upon meticulous examination, it was observed that the pathways of gene expression mutually influence one another, thereby indicating the crucial role played by hsa‐miR‐200c‐3p as a key player in this particular pathway. It is noteworthy that the pathways illustrated in Figure 5 exhibited nodes with a darker color, which points to the presence of significantly enriched gene sets. Moreover, it was observed that the size of these nodes within the network directly corresponds to the size of the gene sets they represent, with larger nodes symbolizing larger gene sets.

**FIGURE 6:**
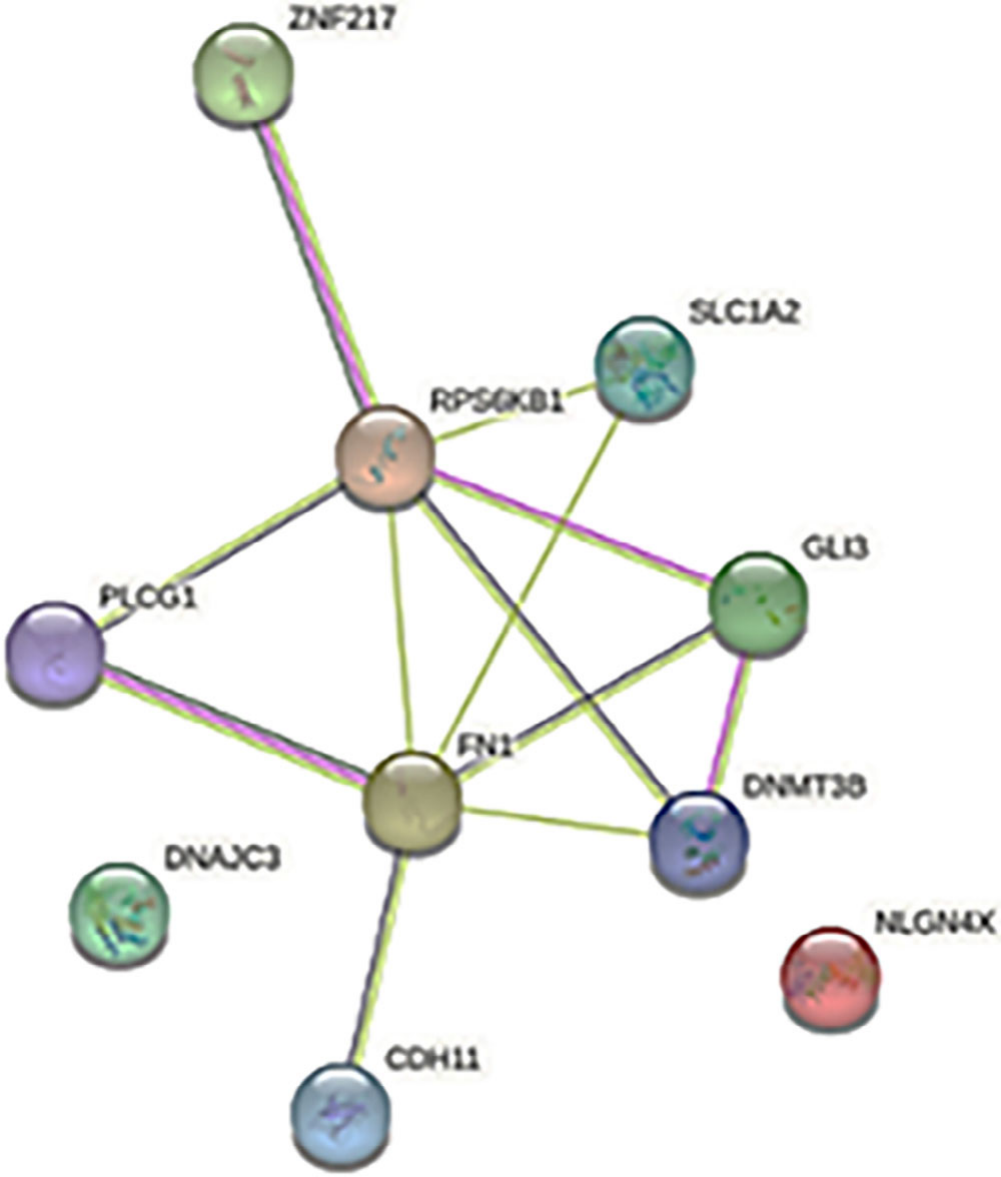
Protein‐protein interaction enrichment network for the whole genes identified in this study by Shiny GO 0.77 software.

Additionally, the thickness of the edges connecting the nodes reflects the degree of overlap between the associated genes as shown in Table 4. Data analysis of protein‐protein interaction between identified genes in this study is illustrated in Figure 6. Based on gene‐gene interaction and PPI data, it can be assumed that there is a high level of interaction between the expressed genes and proteins in different pathways, and that hsa‐miR‐200c‐3p may play a pivotal role in altering the expression of genes that participate in this pathway.

**TABLE 4:** The network dot and edge size for pathways are predicated.

A) Dot size	
Id	Dot size
Proteoglycans in cancer	8.784496053
Pathways in cancer	10.09732203
EGFR tyrosine kinase inhibitor resistance	7.265476457
ErbB signaling pathway	7.265476457
PD‐L1 expression and PD‐1 checkpoint pathway in cancer	7.265476457
Fc gamma R‐mediated phagocytosis	7.265476457
Choline metabolism in cancer	7.265476457
AGE‐RAGE signaling pathway in diabetic complications	7.265476457
HIF‐1 signaling pathway	7.265476457

B) Network edge		
From	To	Width
Proteoglycans in cancer	Pathways in cancer	3.2
Proteoglycans in cancer	EGFR tyrosine kinase inhibitor resistance	2.8125
Pathways in cancer	EGFR tyrosine kinase inhibitor resistance	1.8
Proteoglycans in cancer	ErbB signaling pathway	2.8125
Pathways in cancer	ErbB signaling pathway	1.8
EGFR tyrosine kinase inhibitor resistance	ErbB signaling pathway	5
Proteoglycans in cancer	PD‐L1 expression and PD‐1 checkpoint pathway in cancer	2.8125
Pathways in cancer	PD‐L1 expression and PD‐1 checkpoint pathway in cancer	1.8
EGFR tyrosine kinase inhibitor resistance	PD‐L1 expression and PD‐1 checkpoint pathway in cancer	5
ErbB signaling pathway	PD‐L1 expression and PD‐1 checkpoint pathway in cancer	5
Proteoglycans in cancer	Fc gamma R‐mediated phagocytosis	2.8125
Pathways in cancer	Fc gamma R‐mediated phagocytosis	1.8
EGFR tyrosine kinase inhibitor resistance	Fc gamma R‐mediated phagocytosis	5
ErbB signaling pathway	Fc gamma R‐mediated phagocytosis	5
PD‐L1 expression and PD‐1 checkpoint pathway in cancer	Fc gamma R‐mediated phagocytosis	5
Proteoglycans in cancer	Choline metabolism in cancer	2.8125
Pathways in cancer	Choline metabolism in cancer	1.8
EGFR tyrosine kinase inhibitor resistance	Choline metabolism in cancer	5
ErbB signaling pathway	Choline metabolism in cancer	5
PD‐L1 expression and PD‐1 checkpoint pathway in cancer	Choline metabolism in cancer	5
Fc gamma R‐mediated phagocytosis	Choline metabolism in cancer	5
Proteoglycans in cancer	AGE‐RAGE signaling pathway in diabetic complications	2.8125
Pathways in cancer	AGE‐RAGE signaling pathway in diabetic complications	1.8
EGFR tyrosine kinase inhibitor resistance	AGE‐RAGE signaling pathway in diabetic complications	1.25
ErbB signaling pathway	Yersinia infection	1.25
PD‐L1 expression and PD‐1 checkpoint pathway in cancer	Yersinia infection	1.25
Fc gamma R‐mediated phagocytosis	Yersinia infection	1.25

*Note*: NB: Network Nodes and edges size and edges calculated by Shiny GO 0.77 software.

### 4.4 Prediction and selection of suitable pathway for hsa‐miR‐200c‐3p in AA patients

From the findings of our study, it was observed that hsa‐miR‐200c ‐3p miRNAs significantly exhibited higher levels in patients with AA in comparison to the control group. In order to gain a deeper understanding of the underlying mechanism responsible for the elevated expression of hsa‐miR‐200c‐3p, we employed bioinformatic tools to conduct a comprehensive analysis. Our analysis led us to propose the existence of a unique pathway that could potentially influence the upregulation of hsa‐miR‐200c‐3p expression that contributes to the development of AA.

In line with this perspective, we delved into the exploration of the genes associated with hsa‐miR‐200c‐3p and observed that RPS6KB1 gene appeared to be a more suitable candidate compared to other identified genes in the study, owing to several compelling reasons. Notably, the RPS6KB1 gene exhibited the lowest binding energy of −19.424, which indicates an easier ability for attachment and detachment with hsa‐miR‐200c‐3p. Furthermore, the expression form of RPS6KB1 displayed the highest percentage of AU‐rich region fraction (0.56), which adds to its potential significance. In addition, the binding probability of hsa‐miR‐200c‐3p with RPS6KB1 was found to be 1.00, further strengthening its candidacy (Table 2).

Upon closer inspection of the top 10 pathways, a rather interesting pattern emerged—the PLCG1 gene was found to be present in all of the pathways and played a substantial role in each one. Based on the fact that AA is an immune‐based disease which is our concern here, we found that out of the 10 pathways investigated, there were 4 pathways closely related to immunity and cell signaling, namely, EGFR tyrosine kinase inhibitor resistance, ErbB signaling pathway, Fc gamma R‐mediated phagocytosis, and HIF‐1 signaling pathway (Table 3).

**FIGURE 7:**
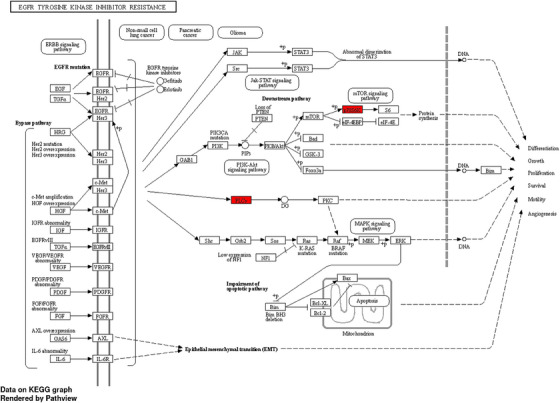
EGFR tyrosine kinase inhibitor resistance pathway genes are highlighted in red (P70S6K is RPS6KB1 gene and PLCy is PLCG1).

It is worth noting that the EGFR tyrosine kinase inhibitor resistance pathway exhibited a significantly higher fold enrichment of 57.7, along with an enrichment FDR of 8.6E‐03. Moreover, both RPS6KB1 and PLCG1 were found to play pivotal roles in the process of tyrosine kinase inhibitor resistance, further solidifying the importance of this pathway (Figure 7).

The selection of the EGFR tyrosine kinase inhibitor resistance pathway was additionally supported by gene‐gene interaction analysis (Figure 5 and Table 4). Notably, the dot size and edge size in the PPI network which is shown in Figure 6 displayed congruent and suitable results in relation to the aforementioned resistance pathway.

Taking into consideration the data obtained from our study, we hypothesize that hsa‐miR‐200c‐3p exerts a direct influence on the EGFR tyrosine kinase inhibitor resistance pathway through its impact on the expression of RPS6KB1 and PLCG1 genes. This intriguing finding opens up new avenues for further investigation into the molecular mechanisms underlying AA and provides valuable insights into potential therapeutic targets for this condition.

## 5 DISCUSSION

It has been reported by several studies that miRNAs have a major contribution to normal functioning and pathological states of HFs.^25^, ^26^ In the current study, we found a statistically significant increase of hsa‐miR‐200c‐3p expression in the blood of the patients’ group in comparison to a control group which indicates the possible potential role of hsa‐miR‐200c −3p in the development and/or progression of AA. To our knowledge, our study is the first study that explores the overexpression of hsa‐miR‐200c −3p in AA and its potential association with the development of this disease.

Other miRNAs and their roles in the pathogenesis of AA have been reported in several studies. Wang et al. reported several miRNAs including the upregulated mmu‐miR‐329, mmu‐miR‐31 which are linked to the uncontrolled development of peripheral regulatory T cells during AA, and mmu‐miR‐155 which is associated with T cell activity inhibitor CTLA4 pathway during the pathogenesis of AA. Also, they mentioned the downregulated mmu‐miR‐100, mmu‐miR‐1, mmu‐miR‐26b, mmu‐miR‐29c, mmu‐miR‐30b and mmu‐miR‐101a.^13^ Of significance, they discovered that under expression of mmu‐miR‐30b and mmu‐miR‐365 resulted in a cytotoxic T lymphocyte‐mediated immune response against HFs by targeting TAP2 gene.^13^

Sheng et al. performed miRNA microarray analysis from blood samples of AA patients against healthy controls, and they showed 36 significant miRNAs.^27^ The most important were miR‐210 and miR‐1246 which marked as the most potent markers for AA because of their significant accuracy. Other reported miRNAs include miR‐185‐5p, miR‐125b‐5p, and miR‐186‐5p.^27^

Two studies documented miRNA named miR‐30b/d as a key role player in the pathogenesis by targeting AA risk genes like STX17, TNXB, and IL2RA. This miRNA is poorly expressed in AA HFs, and it was reported that its upregulation results in an increase in T‐cell response.^28^, ^29^

Interestingly, unique miRNAs namely miR‐150 and miR‐21 were reported by Gulati et al. and suggested to be utilized in novel miRNA‐based therapeutics for AA. This is because of their major implication on delayed‐type hypersensitivity reaction which is reported to a significant role in several autoimmune diseases including AA.^30^

Going back to our study, we found that hsa‐miR‐200c −3p expression has high diagnostic accuracy with a sensitivity and specificity of 80.5% and 96.6%, respectively. This raises a question about its valuable implications for theranostic purposes in AA. Based on this, we decided to find out the genes targeted by hsa‐miR‐200c −3p using multiple target prediction tools like miRWalk, TargetScan, miRDB, and MirTarBase. Also, to determine which KEGG pathways are affected by selected genes and the role of hsa‐miR‐200c −3p on the determined pathway.

This is through conducting fold enrichment analysis and KEGG pathway analysis. As a result, we were able to reveal that PLCG1 and RPS6KP1 were the most relevant genes that play pivotal roles in the pathogenesis of AA. This is because both genes exist nearly in all pathways, indicating their significance. Moreover, RPS6KP1 gene shows the lowest binding energy, the highest percentage of AU‐rich region fraction, and the binding probability of hsa‐miR‐200c‐3p with RPS6KB1 was found to be 1.00. Interestingly, in our study, both genes were found to play crucial roles in EGFR tyrosine kinases resistance pathway, which intern significantly exhibited the highest fold enrichment.

From the standpoint that hsa‐miR‐200c −3p strongly controls the expression of PLCG1 and RPS6KB1, we subsequently indicate the fluence of hsa‐miR‐200c −3p on EGFR tyrosine kinases resistance pathway. This can enlighten the era of the potential theranostic role of this novel miRNA in AA.

Several tyrosine kinase signaling pathways have been implicated in many diseases that carry autoimmune pathophysiology background. This has culminated in the development and approval of several Tyrosine kinase inhibitors and Jak inhibitors for the treatment of autoimmune diseases like rheumatoid arthritis and related inflammatory diseases.^31^, ^32^

EGFR tyrosine kinases are transmembrane tyrosine kinases that recognize various hormones, cytokines, and growth factors. Also, they maintain adult tissue hemostasis including cell proliferation, adhesion, survival, and migration.^33^ Furthermore, the role of EGFR tyrosine Kinase in the stability of the hair cycle is well established.^34^ The defect on this pathway is associated with many immune‐mediated diseases and cancer.^35^ The role of EGFR tyrosine Kinases signaling pathway in inflammatory skin diseases including AA is poorly understood and has not been yet investigated in clinical trials before. Few experimental animal model studies have discussed the involvement of EGFR in the pathogenesis of atopic dermatitis and psoriasis, in addition to its potential organizing role in epidermis.^36^, ^37^, ^38^

Interestingly, it's well‐recognized that many EGFR tyrosine kinase inhibitors have dermatological, specifically hair‐related side effects.^39^ One of the most characteristic side effects is trichomegaly which is accelerated hair growth induced by Cetuximab.^40^ Hypertrichosis of eyelashes is also a striking common feature.^40^, ^41^, ^42^ On the other hand, alopecia as a side effect of EGFR inhibitors has been documented by many studies.^43^, ^44^

## 6 CONCLUSION

Our findings clearly show, and for the first time the significant overexpression of novel miRNA, hsa‐miR‐200c‐3p, in the blood of patients with AA against controls. Further, we were able to indicate the impact of hsa‐miR‐200c‐3p on both PLCG1 and RPS6KP1 genes which intern control EGFR tyrosine kinases resistance pathway that significantly exhibited the highest fold enrichment. The results enlighten the era of the potential theranostic role of this novel miRNA in AA.

## 7 FUTURE RECOMMENDATION

To further validate the findings of this research, an additional study must be conducted using lesional skin biopsies and a larger sample size of patients. This subsequent study should aim to evaluate the expression levels of both PLCG1 and RPS6KP1 genes. In addition to this, the protein levels of PLCG1 and RPS6KP1 genes must be quantified using the highly reliable and widely accepted western blot technique.

## CONFLICT OF INTEREST STATEMENT

The authors declare no conflicts of interest.

## CONSENT TO PARTICIPATE

Informed consent was obtained from all individual participants included in the study.

## ETHICS STATEMENT

This study was performed in line with the principles of the Declaration of Helsinki. Approval was granted by the Ethics Committee of Shaqra University under reference Number: ERC_SU_202300031

## STATEMENT OF RESPONSIBILITY

Abd Elneam AI: Study design, result analysis and writing manuscript and abstract. Al‐Dhubaibi MS: Introduction writing and forming tables & figures. Bahaj SS: Samples collection and writing manuscript. Alhetheli G General supervising, writing discussion section and submission for publication. All authors contributed to the study conception and design. All authors commented on previous versions of the manuscript. All authors read and approved the final manuscript.

## Data Availability

Data available with authors upon request.
