# Correction to “MiR‐200c‐3p as a Novel Genetic Marker and Therapeutic Tool for Alopecia Areata”

**DOI:** 10.1111/srt.70215

**Published:** 2025-08-26

1

AbdElneam AI, Al‐Dhubaibi MS, Bahaj SS, Alhetheli G. MiR‐200c‐3p as a novel genetic marker and therapeutic tool for alopecia areata. *Skin Res Technol*.2024;30:(3):e13639. doi:10.1111/srt.13639.

Following the publication of the article, concerns were raised regarding the poorly defined scientific rationale and the lack of methodological details. This correction has been issued to address these concerns. The missing information, provided below, should be considered as an addition to the relevant sections of the published article.

## 2 Aim of the Study

The selection of miR‐200c‐3p in this study is supported by several previous investigations highlighting the functional relevance of the miR‐200 family in hair and skin biology. Ding et al. (2022) conducted an integrative analysis of the lncRNA–miRNA–mRNA competitive endogenous RNA (ceRNA) network and identified the miR‐200 family as functionally associated with the murine hair follicle cycle, emphasizing its potential regulatory role in hair growth and regeneration processes [45]. Additionally, Chevalier et al. (2019) reported that the miR‐200 family plays a direct role in skin physiology and contributes to the functional impairments observed during skin aging, further supporting its involvement in cutaneous biological processes [46]. Moreover, Hoefert et al. (2018) demonstrated that members of the miR‐200 family coordinately regulate cell adhesion and proliferation during hair morphogenesis, providing mechanistic insights into their role in hair follicle development [47]. Finally, Wu et al. (2021) confirmed the specific role of miR‐200c‐3p in hair‐related structures by showing that it modulates cochlear hair cells under oxidative stress through regulation of Taok1 expression [48].

## 3 RNA Extraction and RT‐PCR

Quantitative amplification was performed using specific primers for mir200c‐3p, which were as follows: forward primer is (GTCTTACCCAGCAGTGT) and reverse primer is (GAACATGTCTGCGTATCTC) [5] and for the housekeeping control hsa‐miR‐16‐5p Forward Primer: 5′‐TAGCAGCACGTAAATATTGGCG‐3’ and Reverse Primer: 5′‐AACGCTTCACGAATTTG‐3′ (criteria of selection of housekeeping control are stable expression across samples, high abundance, minimal interindividual variation, lack of regulation in many conditions, and used previously in several miRNA quantifications [49, 50, 51, 52, 53, 54] employing a Bio‐Rad iCycler [Bio‐Rad Laboratories, Hercules, CA]). Real‐time PCR conditions were set as follows: initial denaturation at 95°C for 3 min, followed by 40 amplification cycles of denaturation at 95°C for 15 s, annealing at 55°C for 30 s, and extension at 60°C for 30 s.

## 4 Discussion

Although Sheng et al. did not identify hsa‐miR‐200c‐3p among the 36 miRNAs reported in their study, the discrepancy between our findings and those of Sheng et al. may be attributed to several methodological and biological factors. Methodologically, Sheng et al. employed microarray‐based profiling, which can differ in sensitivity and specificity compared to our qRT‐PCR–based validation, potentially affecting detection accuracy. Additionally, their study included a limited sample size (*n* = 5), which may reduce statistical power and limit the detection of subtle expression changes. Differences in patient selection criteria—such as focusing on individuals with more severe forms of AA—may also contribute to divergent results. Biologically, miRNA expression is known to vary across populations, sample types, and environmental contexts, all of which could influence the detection of hsa‐miR‐200c‐3p.
